# Characterization of the Epidermis and Cuticle of the Cashew Pseudofruit during Its Development and Maturation

**DOI:** 10.3390/plants12020293

**Published:** 2023-01-08

**Authors:** Magda Andréia Tessmer, Bruno Geraldelli Ribeiro, Ricardo Alfredo Kluge, Alejandra Salvador, Beatriz Appezzato-da-Glória

**Affiliations:** 1 Biological Science Department, Luiz de Queiroz College of Agriculture, University of São Paulo, Piracicaba 13418-900, SP, Brazil; 2 Postharvest Department, Instituto Valenciano de Investigaciones Agrarias, 46113 Valencia, Spain

**Keywords:** *Anacardium occidentale* L., anatomy, greasiness, microscopy, skin

## Abstract

The epidermis and cuticle play an important role in reducing dehydration and protecting the cashew pseudofruit in both the production environment and the postharvest stage. This study analyzes the alterations on the epidermis and cuticle of CCP 76 cashew pseudofruits harvested in five developmental and maturation stages (S1, S2, S3, S4, and S5). The epidermis and cuticle of the samples were analyzed under light microscopy (LM) (quantitative analysis), scanning electron microscopy (SEM), and transmission electron microscopy (TEM). The epidermal cells at S3 reached maximum outer periclinal wall thickness, which reduced during ripening (S4 and S5), while the cuticle increased in thickness during the same period. These changes coincided with the rapid initial growth of the cashew pseudofruit when the epidermis and cuticle need to accompany the expansion of internal tissues. At the ultrastructural level, lipid material is transported via vesicles through the cell wall to the cuticle, increasing its thickness. Epicuticular waxes, previously deposited as plates and globules, began to develop an amorphous shape during maturation. This process possibly occurs due to changes in wax composition that can be related to the development of greasiness on the fruit skin. These findings provide a better understanding of cashew pseudofruit skin, which will aid future studies and strategies to preserve quality during the postharvest stage.

## 1. Introduction

The cashew tree (*Anacardium occidentale* L.) belongs to the Anacardiaceae family [1] and is native to the Amazon region of Brazil [2]. Its reproductive structure is composed of an achene (true fruit) and an edible fleshy portion (pseudofruit) originating from the peduncle [3] of bisexual flowers [1].

Cashew production in Brazil is significant, with 141,418 tons of these nuts being produced, and the country was the world’s largest cashew pseudofruit producer in 2018 with 1,541,010 tons [4]. Among the clones developed by Empresa Brasileira de Pesquisa Agropecuária (EMBRAPA), the early dwarf clone CCP 76 is the most cultivated, due to the high quality of its cashew pseudofruit and its high storage potential [5,6]. 

Cashew pseudofruit is non-climacteric [7], with a fleshy juicy pulp rich in vitamin C and bioactive compounds such as polyphenols and carotenoids, and a pleasant aroma [8,9]. The skin is delicate, with vibrant colors [9], and it plays an important role in protecting cashew pseudofruits. 

The fleshy fruit skin is composed of an epidermis covered by a cuticle and underlying subepidermal layers [10,11]. The epidermis may have one or more cell layers [11,12,13,14] with or without stomata, trichomes of different types, and lenticels (subepidermal origin) [15]. 

The cuticle is a lipophilic layer synthesized by epidermal cells and consists of cutin, cutan, and epicuticular waxes. Cutin is an insoluble matrix composed of C_16_ long-chain lipid polyesters and C_18_ esterified fatty acids, which incorporate amorphous and hydrophobic intracuticular waxes, phenolic compounds, and polysaccharides from cell-wall degradation. Cutan is a lipid biopolymer and may also be present and combined with cutin [14,16,17,18,19]. External to the cutin, epicuticular waxes are crystallized and exhibit crystals with varied morphology and composition [16,20,21].

Along with the cuticle, the epidermis is responsible for important functions during the fruit’s growth, maturation, and postharvest periods. These tissues have a dynamic growth during fruit development and maturation and cover external microcracks caused by environmental factors [22,23,24,25], such as damage caused by solar radiation and temperature and mechanical injuries, while preventing water loss, protecting against pathogen attacks, and regulating gas exchange [13,14,24,26].

Therefore, cuticle preservation is essential to ensure the quality and postharvest potential of the cashew pseudofruit. The cashew pseudofruit undergoes changes in the cuticle, resulting in a greasy appearance after harvest and during cold storage, which can compromise the fruit’s commercial quality. In apples, the development of greasiness during storage has also been identified as a disorder related to changes in cuticular waxes [27,28,29]. ‘Jonagold’ and ‘Cripps Pink’ apples accumulation of more fluid wax constituents led to a solid–liquid phase change, ultimately causing a greasy feeling [28]. 

This study aims to characterize the epidermis and cuticle structure of cashew pseudofruits during development and maturation to improve our understanding of the development of greasiness in this pseudofruit.

## 2. Results

### 2.1. Morphometry and External Color

**Figure 1:**
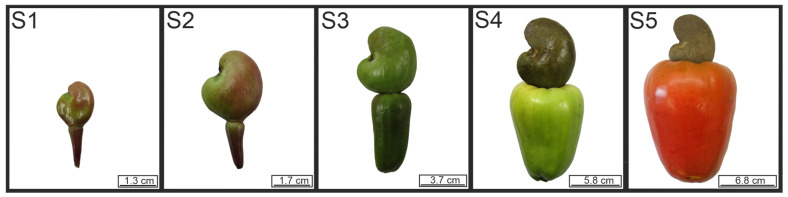
Development and maturation stages of the pseudofruit of CCP 76 cashews.

**Figure 2:**
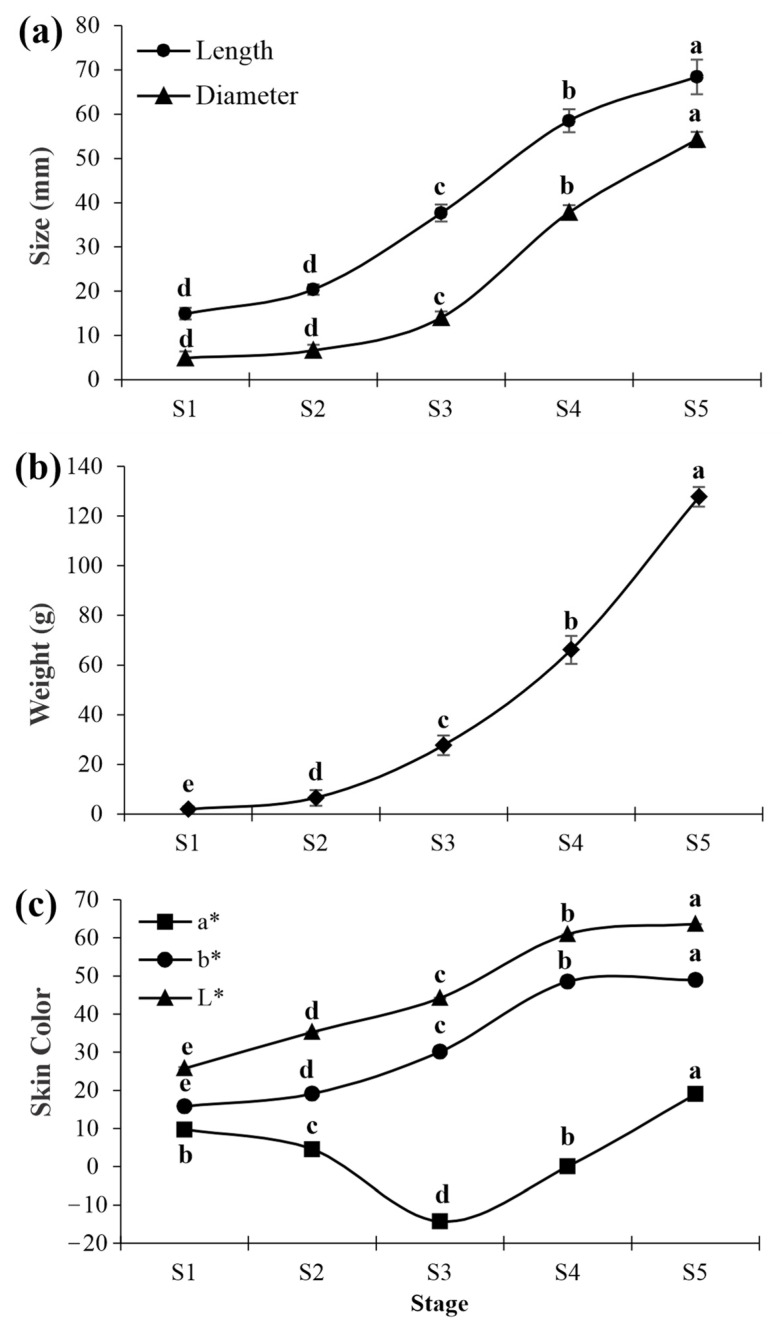
Length and diameter (**a**), weight (**b**), and skin color (**c**) of CCP 76 cashew pseudofruits in five stages (S1, S2, S3, S4, and S5). The means followed by different lowercase letters are significant using Tukey’s test (*p* < 0.05). Vertical bars represent the standard error of the mean. L* (lightness) (dark to light), a* (green to red), and b* (blue to yellow).

Major growth was observed during the developmental and maturation stages of cashew pseudofruits (Figure 1). The pseudofruit’s diameter and length showed a simultaneous increase from S2 onward (Figure 2a), up to a diameter of 54.25 mm and a length of 68.42 mm in S5. In parallel, a gradual increase in the pseudofruit’s weight occurred, with values of 127.80 g in the last maturity stage (Figure 2b).

Likewise, skin color underwent significant changes. In S1 and S2, the pseudofruits were brown and changed to yellow tones in S3 and S4, while in S5 the fruit displayed a marked reddish coloration (Figure 1). These changes were reflected in the evolution of color parameters (Figure 2c). Parameters ‘L’ and ‘b’ exhibited a gradual increase as the postharvest period advanced, with values from 25.75 to 63.61 for ‘L’ and from 15.80 to 48.95 for ‘b’. Parameter ‘a’ obtained values of 48.94 in S1, which lowered slightly in S2. Then, a drastic decrease occurred in S3 and S4 (values of −14.33 and −15.12), which coincided with the color change to green tones. The fruit also exhibited yellow tones. A significant increase in the ‘a’ value was observed when the fruit turned red in S5. 

### 2.2. Structural Characterization of the Epidermis and Cuticle

**Figure 3:**
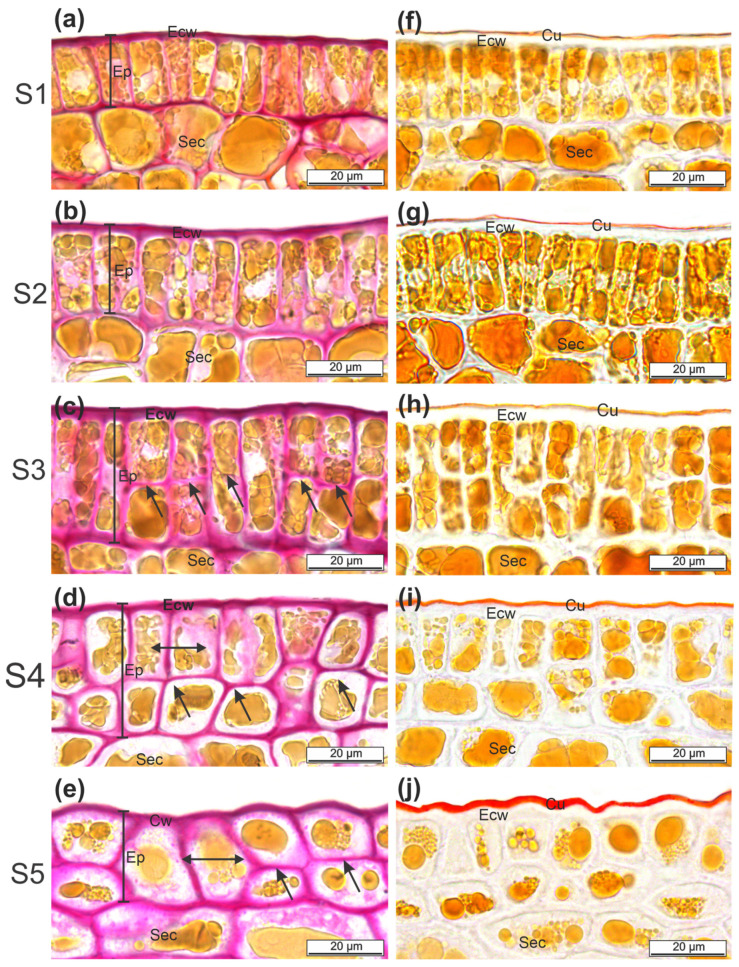
Photomicrographs of cross-sections of CCP 76 cashew pseudofruits in five stages (S1, S2, S3, S4, and S5). (**a**–**e**): Ruthenium red test for pectic substances and polysaccharides showing the cell wall. (**f**–**j**): Positive test for lipids with Sudan IV showing the cuticle. Cell division (black arrow). Cu = cuticle, Ecw = external cell wall, Ep = epidermis, Sec = subepidermal cell.

**Figure 4:**
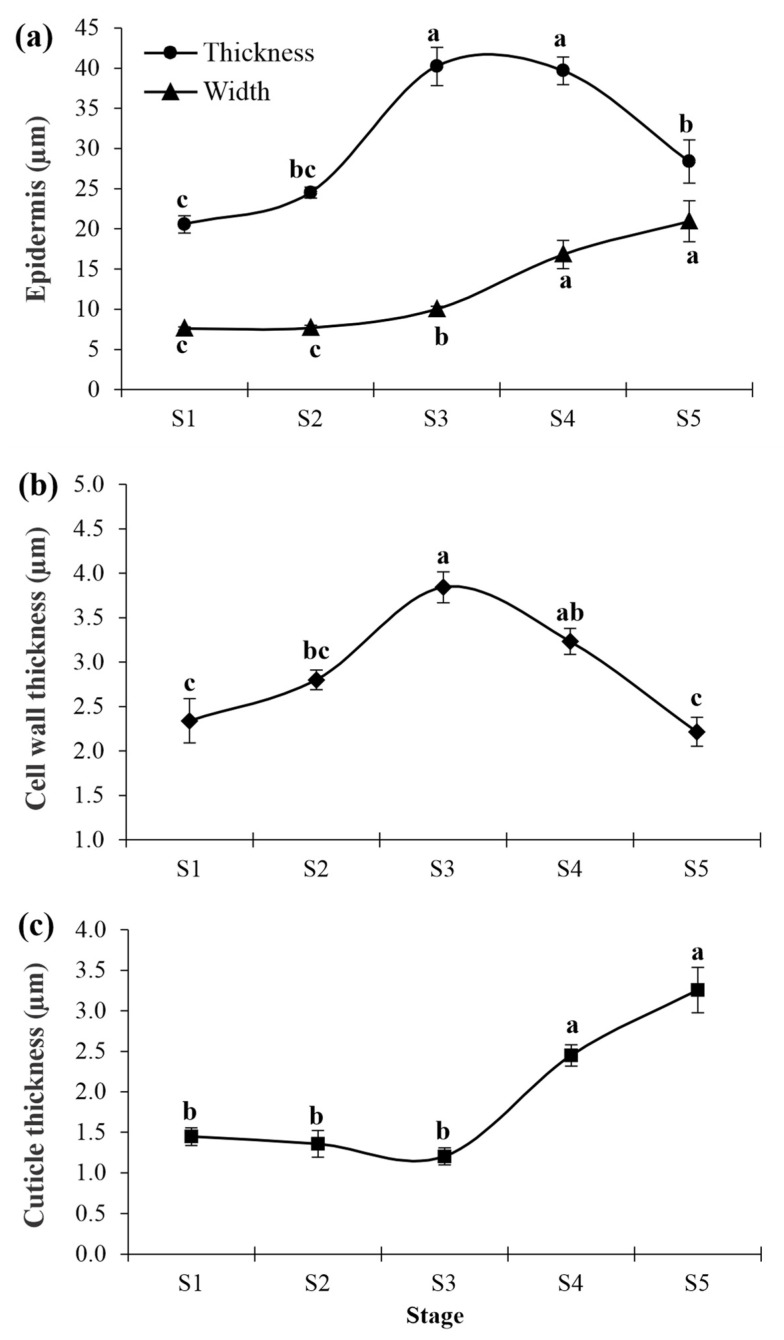
Epidermis thickness and epidermis cell width (**a**), epidermal cell wall thickness (**b**), and cuticle thickness (**c**) of CCP 76 cashew pseudofruits in five development stages (S1, S2, S3, S4, and S5). Means followed by different lowercase letters are significant using Tukey’s test (*p* < 0.05). Vertical bars represent the standard error of the mean.

A cross-section of the cashew pseudofruit in S1 and S2 showed a uniseriate epidermis with rectangular cells (Figure 3a,b), which were 20.55 μm long and 7.59 μm wide (Figure 4a). The height of the epidermis’s cell wall was close to 2.34 μm and 2.80 μm in S1 and S2, respectively, but the differences were not significant. 

In S3, epidermal cells underwent periclinal divisions, resulting in a biseriate epidermis that was more evident in S4 (Figure 3c,d). Consequently, a significant increase in the epidermis’s thickness occurred, with values close to 40.00 μm in S3 and S4 (Figure 4a). The cells resulting from periclinal divisions became shorter and wider. The cell wall thickness of the epidermis increased to 3.84 μm in S3, which was 61% higher than in S1, and then decreased slightly in S4 (Figure 4b).

In S5, the two layers of epidermal cells were flattened (Figure 3e), with an irregular shape, which decreased the epidermis’s thickness to 28.70 μm. Cell width increased slightly, but without differences from the cells from S4. A significant reduction to 2.21 μm occurred in the cell wall thickness. In all stages analyzed, the epidermal and subepidermal cells presented one vacuole or more with phenolic content (Figure 3).

Sudan IV staining permitted assessing the cuticle thickness (Figure 3f–j). The cashew pseudofruits from S1, S2, and S3 showed a continuously thin cuticle layer without flange formation between the anticlinal walls of the epidermal cells (Figure 3f–h). No differences were found in cuticle thickness in these early stages, with values between 1.20 μm and 1.40 μm (Figure 4c). Nevertheless, gradual thickening occurred from S4 (Figure 3i,j) up to values of 3.25 μm in S5 (Figure 4c).

### 2.3. Structural Characterization of the Pseudofruit’s Surface 

**Figure 5:**
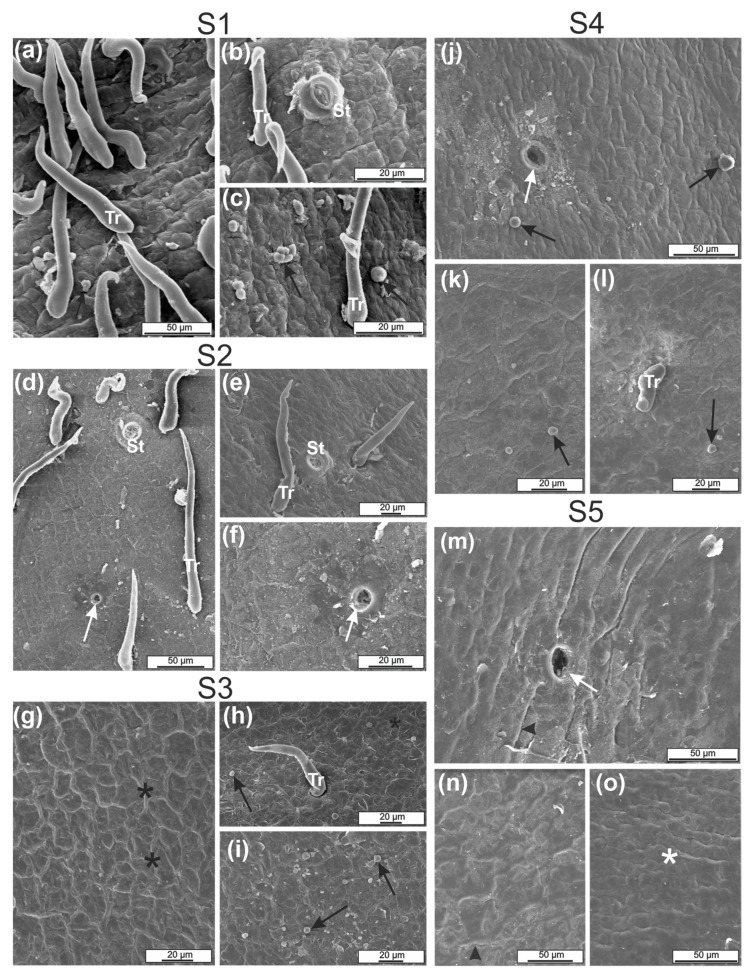
Scanning electron micrographs of the cuticle of CCP 76 cashew pseudofruits in five stages (S1, S2, S3, S4, and S5). S1 (**a**–**c**), S2 (**d**–**f**), S3 (**g**–**i**), S4 (**j**–**l**), and S5 (**m**–**o**). Globular wax (black arrows), epicuticular wax ornamentation (* black), altered epicuticular wax ornamentation (* white), superficial microcracks (arrowheads), and trichome scars (white arrows). St = stomata, Tr = trichome.

Scanning electron microscopy (SEM) evaluation of the pseudofruit’s rind surface revealed notable differences among fruit cuticles at several maturation stages. In S1, a very high density of unicellular non-glandular trichomes occurred (Figure 5a–c). The skin surface was rough, with very pronounced sunken areas due to irregular plate-shaped epicuticular wax. Some globular wax was also dispersed over the cuticle. Stomata were clearly visualized (Figure 5b).

In S2, fewer trichomes were seen because of their breakage, with visible scars on the surface. Irregularly shaped wax platelets were seen (Figure 5d–f). In S3, trichomes were rarely found. Globular wax was observed and epicuticular waxes started to present topographic ornamentation on the epidermal cell walls as a periclinal elongation (Figure 5g–i).

In S4, some fragments of trichomes were seen. At the broken trichome sites (scars), apertures reaching deeper layers, devoid of cuticles, were visible. Globular wax was maintained, the topographic ornamentation became less evident, and some regions displayed small whitish wax crystals. Some shallow surface microcracks were observed (Figure 5j–l). 

In S5, the epicuticular wax looked amorphous, suggesting structural and biochemical changes. Stomata became sparser due to the maximum growth of the mature pseudofruit, scars were left by the ablation of trichomes, and some microcracks were clearly observed on the cuticle (Figure 5m–o). 

### 2.4. Ultrastructural Analysis of the Epidermis and Cuticle

**Figure 6:**
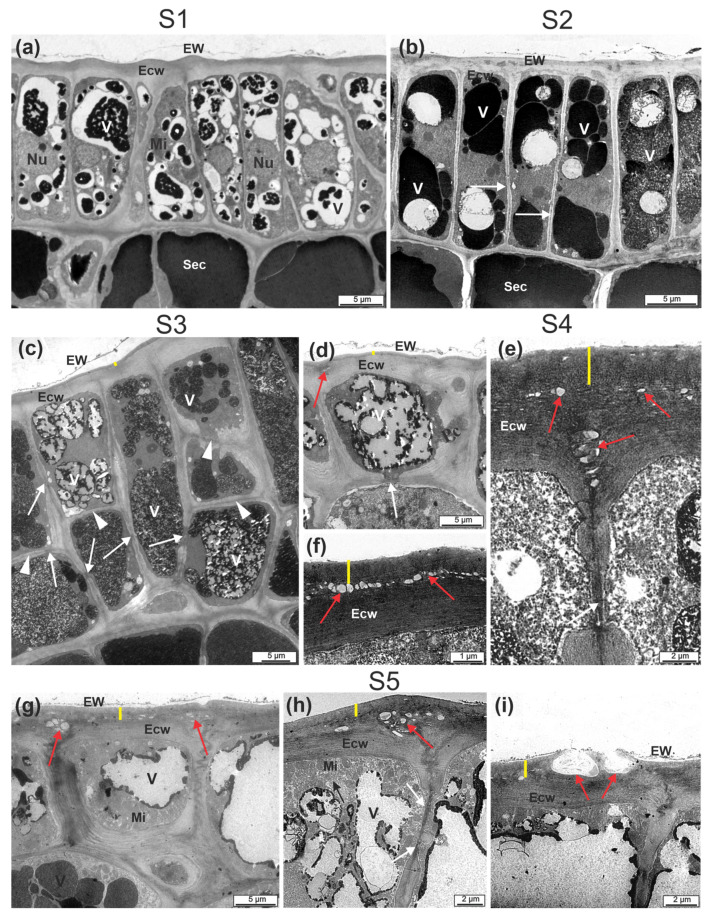
Electron transmission micrographs of the skin of CCP 76 cashew pseudofruits in five stages (S1, S2, S3, S4, and S5). S1 (**a**), S2 (**b**), S3 (**c**), S4 (**d**–**f**) and S5 (**g**–**i**). (**a**) Uniseriate epidermis and subepidermal cells with vacuoles filled with an electron-dense content. (**b**) Taller cells and more filled vacuoles; plasmodesmata in the anticlinal walls. (**c**) Biseriate epidermis with a thicker outer periclinal wall and vacuole with a granular content. In stages S4 (**d**–**f**) and S5 (**g**–**i**), the delimitation between the cuticle and the more evident cell wall is observed. A loose arrangement of the microfibrils of the external wall is observed, along with the presence of vesicular-looking structures when the content was released on the surface (**i**). Red arrows = spaces or vesicles; white arrows = plasmodesmata. Cuticle (Cu) = yellow dash, Ecw = external cell wall, EW = epicuticular wax, Mi = mitochondria, Nu = nucleus, Sec = subepidermal cell, V = vacuole.

Transmission electron micrographs revealed that the epidermal cells in S1 showed an evident nucleus, many vacuoles were partially filled with an electron-dense content, and the periclinal cell walls were thin (Figure 6a). In the subepidermal cells, vacuoles were filled with an electron-dense content. In S2, the epidermal cells exhibited vacuoles filled with more electron-dense content. Plasmodesmata were observed on the anticlinal walls (Figure 6b).

In S3, the periclinal walls became evident and two overlapping cells were observed that formed a biseriate epidermis (Figure 6c). In this stage, the outer periclinal wall became thicker, the anticlinal walls showed more plasmodesmata, and the vacuolar content looked granular.

In stages S4 (Figure 6d–f) and S5 (Figure 6g–i), significant ultrastructural changes in the epidermal cells occurred compared to the previous stages. The boundary between the cuticle and the cell wall became more evident (Figure 6e–h). Microfibrils of the external cell wall started to display a looser arrangement, where vesicular structures with a low density content were observed, especially in stage S5, when the release of the vesicular content on the surface occurred.

## 3. Discussion

### 3.1. Adaptive Changes in the Epidermis during Cashew Pseudofruit Development

The CCP 76 cashew pseudofruit grew slowly in the initial stages (S1 and S2); however, its growth increased quickly after 30 days until its cycle was completed in about 50 days, confirming the descriptions for this species [6]. The rapid growth occurred due to the increase in both length and diameter, which were associated with weight gain from stage S3, when profound epidermal changes occurred. 

The epidermis was uniseriate with palisade-like cells until S3. From this stage, it became biseriate, with shorter and wider cells. The literature data show two differentiation possibilities for the epidermis throughout the development of fleshy fruits, that is, fruits with a uniseriate epidermis and fruits in which epidermal cells divide into more advanced development stages and become bi- or multiseriate. ‘Cascada’ tomato [30,31] and ‘Keitt’ mango [10], as well as ‘Bluecrop’, ‘Earliblue’, and ‘Patriot’ blueberries [32,33] present a uniseriate epidermis until maturation. The bi-multiseriate epidermis pattern occurs in ‘Clapp’s Favorite’ and ‘Conference’ [12] pears, ‘Ligol’ [11], ‘Jonagold’, and ‘Szampion’ [34] apples; and also in ‘Sweet Common Prune’ and ‘President’ plums [35]. Nevertheless, the epidermis pattern can differ between cultivars of the same species, as in the case of the plum, and is uniseriate in ‘Bluefre’ [35].

During the maturation period of the pseudofruit (S4 and S5), the epidermis’s thickness reduced due to increased cell width and narrow external cell wall thickness. In pear cultivars ‘Clapp’s Favorite’ and ‘Conference’, an epidermis formation pattern similar to that of the cashew pseudofruit occurred, that is, with periclinal divisions and stratification, and subsequent reduction in cell height up to 40% during maturation [35]. Epidermal cell division and changes in shape seem to be an adaptive mechanism for the rapid growth of the pseudofruit, which showed in its dynamic ability to follow the expansion of internal tissues and prevent the formation of microcracks, which were not commonly observed in the pseudofruits analyzed in our study. Despite epidermal changes, no wax deposition occurred between the anticlinal walls of the cells, as observed in other fruit such as ‘Keitt’ mangoes [10,22] and ‘Gala’ and ‘Galaxy’ apples [22], confirming that epidermal cells did not separate until maturation.

Epidermal and subepidermal cells showed many vacuoles of different sizes with phenolic content in all the stages analyzed. The vacuolar content, along with pigments in chloroplasts (chlorophylls and carotenoids), caused changes in the pseudofruit’s color index. Tezotto-Uliana et al. (2018) [9] found 0.04 g kg^−1^ of anthocyanins in cashew pseudofruit skin harvested with 70% orange-red skin, which was responsible for the increased color. The same authors also identified 0.22 g kg^−1^ of hydrolyzable polyphenols and 20–24 g kg^−1^ of proanthocyanidins in the skin and 0.7–1.0 g kg^−1^ in the pulp, indicating that the skin was highly astringent. 

In the cashew pseudofruit’s epidermis, non-glandular trichomes and stomata were distributed very densely. The removal of trichomes left scars that remained on the epidermis throughout development until the fruit’s maturation. The presence of trichomes in the early developmental stages and during scar formation has also been recorded in some apple cultivars, such as ‘Gala’, ‘Galaxy’, ‘Jonagold’, and ‘Szampion’ [34]. Trichomes are possibly involved in protecting the floral receptacle and in preventing infections and pest attacks in the early developmental stages of the cashew pseudofruit [34]. Thus, trichomes appear only in the initial development stages of the pseudofruit.

Stomata were already formed in S1 with different degrees of opening. The coating and its natural openings determine the diffusion of gases (respiration) and transpiration in fruit [36]. Therefore, the stomata and scars left by trichomes on the cashew pseudofruit’s epidermis, as observed in the maturation stage (S5), could affect postharvest qualitative characteristics.

### 3.2. Particular Deposition Pattern and Greasiness Development of the Cuticle

The cuticle has particularities in fruit of all species; however, Lara et al. (2015) [16] report two cuticle deposition trends. First, cuticle deposition ceases at a certain developmental stage, usually between growth and ripening, subsequently reducing during wax biosynthesis and cuticle thickness, as reported for the sweet cherry. Second, biosynthesis and cuticle thickness continuously increase until ripening, as verified in tomatoes. The cashew pseudofruit does not follow either of these trends, as its cuticle thickness remained stable with the expansion of periclinal walls in S3 (1.20 μm), along with a subsequent 44.48% increase in maturity. The increase in cuticle thickness during maturation seems to be a form of compensation or protection to reduce the occurrence of epidermal thickening.

SEM observations showed few superficial microcracks in the cuticle, possibly due to increased wax biosynthesis during maturation. Microcracks occur on the cuticle because of its inability to accompany fruit growth [23,25], as well as wax biosynthesis during ripening [24] and cold storage [22,37]. However, the analysis performed on the skin surface showed that wax structure and composition altered, which rendered an amorphous aspect by the end of the maturation stage. This amorphous aspect coincided with the development of greasiness observed in pseudofruit in the preliminary experiments performed after harvest and during cold storage. Additionally, cashew pseudofruit showed increased luminosity (lightness) which, according to Lara et al. (2014) [24], indicates a biosynthesis increase in alcohols in cuticle composition.

Greasiness has been recently investigated as a disorder identified on apple skin. Greasiness is known to be related to the accumulation of liquid cuticle constituents, such as esters of oleate and linoleate of (E,E)-Farnesol and short-chain alcohols (C3–C5) [28,38,39]. Significant increases in gene expression related to biosynthesis and export of fluid wax constituents during the ripening and postharvest stages have also been identified [38]. Environmental conditions, such as temperature and solar radiation [40], are associated with specific characteristics of species and varieties [16]. In ‘Gala’ and ‘Galaxy’ apples, changes in wax crystals that resemble fatty agglomerates have been observed during storage in a controlled atmosphere [22].

### 3.3. Vesicles in Epidermal Cells and Cuticle May Be Related to Wax Transport 

In the early stages S1 and S2, epidermal cells have thin anticlinal walls, evident nuclei, and small vacuoles with an electron-dense content. In S3, cells of both epidermal layers exhibit a thickening of the anticlinal and periclinal walls, along with the presence of numerous plasmodesmata. The role of plasmodesmata in mediating macromolecule traffic is well-known in the vegetative organs of plants [41,42], but little is known about their role in fruit epidermises. The plasmodesmata verified in the epidermal cells of the cashew pseudofruit indicate the possible transport of substances to neighboring cells. Plasmodesmata have also been found in the epidermis of mature ‘Szampion’ apples [23].

The mechanism of biosynthesis and wax deposition is not fully understood in fruit [16,17,24]. According to Tafolla-Arellano et al. (2013) [17], wax biosynthesis consists of three stages: synthesis of fatty acids with 16–18 carbons in chloroplasts [43,44]; transport of these fatty acids to the endoplasmic reticulum for elongation, with the formation of very long-chain fatty acids (alcohols, esters, aldehydes, alkanes, and ketones) [18]; and transport of lipids outside the cell wall of epidermal cells, possibly to help transporters in the plasma membrane and epidermal cell wall. 

During the cashew pseudofruit maturation process, many vesicles cross the outer wall of epidermal cells, and the contents of these vesicles are released onto the cuticular surface. Similar electron micrographs have been recorded on citrus petal epidermis by Marques et al. (2016) [45], who related these vesicles to the transport and release of lipid material in the cuticle. The thickening of the cuticle occurred during the developmental stage of the cashew pseudofruit. Konarska (2013) [12] also verified the presence of numerous structures between the cuticular layer and the cuticle itself in pear varieties and suggested that they were spaces or vesicles. However, the factors regulating the location of fatty acid precursors and cutin synthesis, as well as the mechanisms of transport and deposition of cutin components, are still unclear. Therefore, further studies are needed.

## 4. Materials and Methods

### 4.1. Plant Material 

Early dwarf cashew CCP 76 clones (*Anacardium occidentale* L.) were obtained in 2017 from an orchard in the municipality of Artur Nogueira, São Paulo State, Brazil (22°34′23″ south, 47°10′21″ west, 588 m altitude). The complete development of cashew apples on the plant requires 50 days on average [6]. Five cashew apple stages (S1, S2, S3, S4, and S5) (Figure 1) were harvested; one cashew apple was collected per plant, in the middle region and around the crown of the plant, and standardized by size (height and diameter), weight, and skin color. Cashew apples were sent to the Luiz de Queiroz College of Agriculture (ESALQ-USP), where they were sanitized with sodium hypochlorite (5 g L^−1^ of StartClor^®^) for 10 min and dried at room temperature. 

For each stage, four replicates of three cashew pseudofruits (12 units of different plants) were used to determine both morphometry and skin color. Moreover, three cashew pseudofruits from different plants (four replicates per sample) were used for the analysis of skin structure under light microscopy (LM), scanning electron microscopy (SEM), and transmission electron microscopy (TEM).

### 4.2. Morphometry Parameters

The length and diameter of the median region of the cashew pseudofruits were determined with a digital caliper (Digimess, model 100.174BL, São Paulo, Brazil). Weight (cashew pseudofruit and nut) was established on a precision scale (Gehaka BK8000, São Paulo, Brazil).

### 4.3. Skin Color

The cashew pseudofruits’ skin color was evaluated by a digital colorimeter (CR-400, Konica Minolta Inc., Tokyo, Japan) using the CIELAB color space system and calibration at D65: the three-dimensional components were L* (lightness) (dark to light), with variation between −100 and +100, and a* (green to red) and b* (blue to yellow), with variation between −60 and +60. The readings were performed in four regions of the cashew pseudofruit.

### 4.4. Light Microscopy (LM)

Transversal skin samples from the equatorial regions of the three cashew pseudofruits were fixed in Karnovsky’s solution [46], subjected to a vacuum pump to remove air from the intercellular spaces, dehydrated in increasing ethyl series up to 100% ethanol, and infiltrated in hydroxy-ethyl-methacrylate (Leica historesin^®^, Heraeus Kulzer, Hanau, Germany) to make blocks. The blocks were sectioned in a rotary microtome (RM 2045, Leica Biosystems, Heidelberg, Germany) at 5–7 μm thickness. Sections on slides were stained with ruthenium red for pectins and polysaccharides [47] and mounted with synthetic resin (Entellan^®^, Merck, Darmstadt, Germany) to analyze the epidermis and its outer cell walls. Sudan IV was used to detect lipids to delimit the cuticle [48]. Digital images were taken under a microscope (DM LB, Leica Microsystems, Wetzlar, Germany) attached to a Leica DC 300 F video camera using LAS 4.0 software (Leica Microsystems, Wetzlar, Germany). 

Four images were taken per sample, with six measurements on each image to measure the thickness, width, and height of the outer wall of epidermal cells and cuticle thickness, totaling 72 measurements per stage. Measurements were taken using ImageJ^®^ software (Wayne Rasband at the National Institutes of Health, USA). 

### 4.5. Scanning Electron Microscopy (SEM)

Skin samples from the equatorial regions of the three cashew pseudofruits were fixed in Karnovsky’s solution [46] and dehydrated in increasing ethyl series from 10% to 100% for 10 min. Then, the samples were dried to the critical point (EM CPD 300, Leica, Wien, Austria) with liquid CO_2_ [49], mounted on stubs, and metallized with an 80 nm gold layer for 220 s in an evaporator (MED 010 Balzers, Carlsbad, USA). The samples were observed under a scanning electron microscope (JSM-IT300, JEOL, Tokyo, Japan) operating at 20 kV to analyze the cuticle surface and digitalize electron micrographs.

### 4.6. Transmission Electron Microscopy (TEM)

Skin transversal samples from the equatorial regions of the three cashew pseudofruits were fixed in Karnovsky’s solution with modifications (2.5% glutaraldehyde, 2.5% paraformaldehyde, and 0.05 mM CaCl_2_ in sodium cacodylate buffer (0.1 M, pH 7.2)) [46] for 48 h. The samples were post-fixed in 1% osmium tetroxide for 2 h, dehydrated in increasing acetone series up to 100%, and infiltrated in resin (Spurr, Electron Microscopy Sciences, Hat Field, PA, USA). The blocks were sectioned in the ultramicrotome (UC6, Leica, Vienna, Austria). Sections were mounted on 200-mesh copper screens and contrasted with 5% uranyl acetate and 2% lead citrate for 30 min in each step [50]. Observations and electron micrographs of the epidermis and cuticle were performed using a transmission electron microscope (JEM 1011, JEOL, Akishima, Japan) with a coupled Gatan 830 J46W44 video camera operating at 60 Kv.

### 4.7. Statistical Analysis

Data were submitted to analysis of variance (ANOVA) and the means were compared using Tukey’s test (*p* < 0.05). The statistical software used was SASM-Agri [51].

## 5. Conclusions

In conclusion, this is the first comprehensive study of the epidermis and cuticle changes that take place during the development and maturation of cashew pseudofruits. The ultrastructural analyses demonstrated that lipid material is transported via vesicles through the cell wall to the cuticle, increasing its thickness. Epicuticular waxes, previously deposited as plates and globules, start to develop an amorphous shape during maturation. This process possibly occurs due to changes in wax composition and leads to the development of greasiness on the fruit skin, which is related to the preservation of cashew quality during the postharvest process.

## Data Availability

The data presented in this study are available upon request from the corresponding author.
